# Supplementary material for: Development of chemokine network inhibitors using combinatorial saturation mutagenesis
Source: Commun Biol. 2025 Apr 3;8:549. doi: 10.1038/s42003-025-07778-6 (PMC11969024; doi:10.1038/s42003-025-07778-6)
Supplement: Supplementary file 4 — Supplementary Data 2 [file 42003_2025_7778_MOESM4_ESM.zip › SupplementaryData3/scripts/xattune_dr.tar!/./script/analysis/JK/JK_E151_CCL19_ATC_plot.pdf]

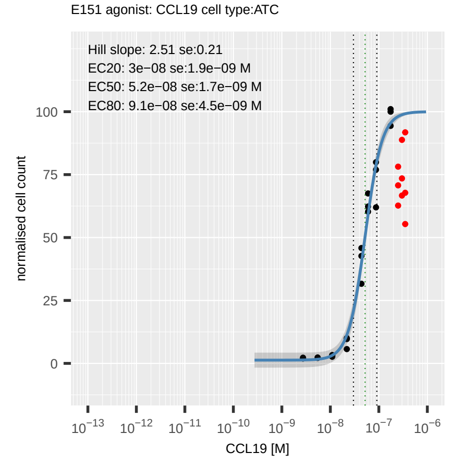

E151 agonist: CCL19 cell type:ATC
Hill slope: 2.51 se:0.21
EC20: 3e−08 se:1.9e−09 M
EC50: 5.2e−08 se:1.7e−09 M
EC80: 9.1e−08 se:4.5e−09 M
normalised cell count
0
25
50
75
100
10^{-13}
10^{-12}
10^{-11}
10^{-10}
10^{-9}
10^{-8}
10^{-7}
10^{-6}
CCL19 [M]
